# Surgical Bureau of I. H. A.

**Published:** 1887-05

**Authors:** 


### THE SURGICAL BUREAU OF THE INTERNATIONAL HAHNEMANNIAN ASSOCIATION.

We are in receipt of a letter from Professor E. Carleton, M. D., of New York, in which he laments that the Surgical Bureau of the Association has not had the support it merits. Indeed, it has been very much neglected. Cases of surgical diseases cured homœopathically have been sent to the Bureau of Clinical Medicine and elsewhere. This should not be. The members of the International Hahnemannian Association should bear in mind that the surgical bureau is of especial importance, in view of the fact that surgery is popularly supposed to be beyond the pale of our school. How much eclectic teaching is given in surgical matters to the students of our colleges! It is our duty to combat this teaching by exhibiting as many cases treated by the Hahnemannian method as possible. Many interesting and instructive cases might be reported. A generous support to this Bureau should be accorded by sending in such cases; free discussions of them should follow that the weak-kneed among us may be encouraged.

Dr. Carleton assures us that the programme of the next meeting will be so amended as to give surgery more prominence than it has had hitherto. Now, let the members come prepared with papers, read them themselves, and join in discussion of the contributions of others. All are interested in making the meeting a success.

